# Estrogen and EGFR Pathways Regulate Notch Signaling in Opposing Directions for Multi-Ciliogenesis in the Fallopian Tube

**DOI:** 10.3390/cells8080933

**Published:** 2019-08-19

**Authors:** Maobi Zhu, Tomohiko Iwano, Sen Takeda

**Affiliations:** Department of Anatomy and Cell Biology, Graduate School of Medicine, University of Yamanashi, 1110 Shimo-Kateau, Chuo, Yamanashi 409-3898, Japan

**Keywords:** motile cilia, ERβ, differentiation, DLL1, high-grade serous ovarian cancer

## Abstract

The lumen of the fallopian tube (FT) is lined with columnar epithelium composed of secretory and ciliated cells, both of which are important for reproduction. However, the molecular mechanism regulating cell fate remains controversial. In this study, we established a primary culture system using porcine fallopian tube epithelial cells (FTECs) to study the differentiation mechanism. We found that estrogen promoted the differentiation of multi-ciliated cells (MCCs) through estrogen receptor β, following the reduction of DLL1, a ligand of Notch. Meanwhile, epidermal growth factor (EGF), a regulator of epithelial homeostasis and differentiation, suppressed ciliogenesis by the activation of Notch signaling. However, the estrogen pathway did not affect the activation of the EGF pathway. Taken together, the differentiation of MMCs in FT depends on the balance of EGF and estrogen signaling, either of which inhibits or stimulates the Notch signaling pathway respectively.

## 1. Introduction

The lumen of the fallopian tube (FT) is lined with a columnar epithelium composed of secretory and ciliated cells, both of which have critical roles in conditioning the epithelial surface for efficient reproduction [1]. The secretory cells produce mucous fluid, and the flow derived from the motile cilia of ciliated cells facilitates the transport of gametes. The epithelial environment is altered during the menstrual cycle. In particular, as the streams of ovarian steroid hormones, such as progesterone and estrogen, change dramatically, various signaling pathways change the condition of fallopian tube epithelial cells (FTECs). Previous studies showed that progesterone and estrogen receptors are expressed in the fallopian tube epithelium (FTE) and regulate the differentiation of FTECs [2,3]. In particular, estrogen (E2) has a role in the induction and maintenance of the mature FTE [4,5]. Furthermore, Donnez et al. suggested the relationship between the generation of MCCs and E2 administration in postmenopausal women [5]. However, the exact molecular pathway regulating the differentiation of each cell type in the FTE remains to be determined.

For the molecular mechanism in the differentiation of ciliated cells and secretory cells, the trachea, which has a similar epithelial structure to that of FTE, has been a representative model. Several studies have shown that Notch signaling controls the equilibrium of ciliated and secretory cells. In the developing airway, Notch activation is sufficient to drive secretory cell formation at the expense of ciliated cells [6], whereas inhibition of Notch signaling leads to an increase in the number of ciliated cells and a concomitant decrease in secretory cell generation [7]. Furthermore, the epidermal growth factor (EGF) pathway also regulates the differentiation of tracheal epithelial cells in concert with Notch signaling [8]. Therefore, the downregulation of the EGF pathway results in the increase of MCCs [9]. 

Increasing lines of recent evidence have shown that the FT may be an origin location for high-grade serous ovarian cancer (HGSC), based on the similarity of gene mutations between HGSC and FTECs, as well as the similar morphological and antigenic features [10,11,12]. Taking the factor into account that Notch signaling is involved in carcinogenesis, elucidation of the molecular mechanisms linking Notch to estrogen signaling would be important. We first aim to understand the molecular pathways in the normal construction of FTE. Although a recent paper described how the inhibition of Notch promoted the differentiation of human MCCs [13], the relationship among other signaling pathways has not been studied. In this study, we focus on the interrelationship among E2, EGF, and Notch pathways, in terms of the differentiation of MCCs, by utilizing the primary culture system of porcine FTECs. 

## 2. Materials and Methods

### 2.1. Cell Culture Medium

The composition of media was modified from that of a previous study for culturing FTECs (17). “Expansion medium” was used when the initial cell harvests were plated. “Basal medium” was used to support growth and to induce differentiation. The basal medium is DMEM/Ham’s F-12 medium (#042-30795, Wako, Chuo, Osaka, Japan) supplemented with 1% GlutaMAX (#35050061, Thermo, Waltham, MA, USA), 2% B27 (#17504-044, Thermo), 1 mM nicotinamide (#72340, Sigma-Aldrich, St. Louis, Mo, USA), 0.5 µM transforming growth factor beta (TGF β) receptor kinase inhibitor IV (#SB431542, Wako), and 10 ng/mL human EGF (PHG0311, Thermo). Unless otherwise specified, EGF was included in the basal medium. For the expansion medium, we added 5 µM Rho-associated coiled-coil containing kinase (ROCK) inhibitor (#030-24021, Wako), 100 ng/mL human fibroblast growth factor (FGF, #100-26, Peprotech, Rocky Hill, NJ, USA), 100 ng/mL human Noggin (#120-10C, Peprotech), 50 ng/mL Wnt-3a (#5036-WN-010, R&D systems, Minneapolis, MN, USA), and 125 ng/mL R-Spondin 2 (#3266-RS-025, R&D systems) into the basal medium. 

To examine the effect on differentiation, cells were treated with reagents as follows: β-estradiol (#E4389, Sigma-Aldrich); diarylpropionitrile (DPN, #1428-67-7, Sigma-Aldrich), which is a synthetic, nonsteroidal and 70-fold more selective for estrogen receptor β (ERβ) over estrogen receptor α (ERα); PHTPP (#805239-56-9. Sigma-Aldrich), which is a synthetic, nonsteroidal antagonist of ERβ and displays 36-fold selectivity over ERα; propylpyrazole triol (PPT, #263717-53-9, Cayman Chemical, Ann Arbor, MI, USA), which is a synthetic, nonsteroidal agonist of ERα with 400-fold selectivity over ERβ; G-1 (#881639-98-1, Cayman Chemical), which is a nonsteroidal, high-affinity, selective agonist of GPR30; DAPT (#208255-80-5, Cayman Chemical), which is an inhibitor of γ-secretase and indirectly inhibits the Notch signal; and gefitinib (#184475-35-2, Wako), which is the first selective inhibitor of epidermal growth factor receptor (EGFR) tyrosine kinase domain by binding to the adenosine triphosphate (ATP)-binding site of the enzyme.

### 2.2. Isolating Primary FTECs 

Porcine fallopian tubes were collected from healthy sows at a local slaughterhouse. The tissues were immersed in ice-chilled DMEM/Ham’s F12 medium and transferred to the laboratory within 30 min. They were washed with Phosphate-buffered saline (PBS) and opened longitudinally to expose the mucosal surface. The epithelium was incubated with 100 U/mL collagenase type IV (Thermo, 17104-019) and 10 µg/mL DNase I (Sigma-Aldrich, 9003-98-9) for 90 min at 37 °C. After that, the epithelial surface was scraped with a cell scraper to collect the epithelial layer. The epithelial sheets were rinsed with PBS and incubated with trypsin (Nacalai, 32777-44, Tokyo, Japan) for 15 min at 37 °C. Subsequently, cell suspension was filtered with a cell strainer (Falcon, 100 µm pore #352360, New York, NY, USA) to remove the undigested aggregation. The collected cells were centrifuged and re-suspended in expansion medium supplemented with 10% fetal bovine serum (FBS), followed by seeding onto a collagen type I coated plastic dish and incubated at 37 °C in 5% CO_2_–conditioned and humidified incubator. One or two days later, after the FTECs attach to the dish, the medium was replaced with expansion medium without FBS. The FTECs were passaged several times on mitotic-inactivated mouse embryonic fibroblasts in the expansion medium. The FTECs were cryopreserved for long-term storage in liquid nitrogen.

### 2.3. Induction of the Differentiation of FTECs in the Air–Liquid Interface (ALI) Culture 

FTECs (1 × 10^5^) were seeded on semipermeable membrane supports (Transwell 0.4 µm pore size; Corning #3470, New York, NY, USA) coated with collagen type I in the basal medium until transition into the ALI culture. Two days after seeding, the apical medium was removed from the upper chamber to open cell surface to the air (ALI day 0). The medium in the bottom chamber was changed to reagent-added medium to induce ciliogenesis. The medium was changed every 3 days.

### 2.4. Immunofluorescence Staining

FTECs cultured on glass coverslips and on transwells were briefly washed with PBS and fixed in 4% paraformaldehyde (PFA) for 10 min at room temperature (RT). The transwell membrane with cells was cut after fixation. Cells were washed with PBS three times, permeabilized with 0.1% Triton X-100 and blocked with 5% goat serum in PBS for 30 min at RT. Cells were then incubated with primary antibodies (Table S1) diluted in blocking solution overnight at 4 °C. After washing, cells were incubated with secondary antibodies (Table S1) for 1 h at RT. Cells were counterstained with DAPI (Thermo, 62248) and subsequently mounted using ProLong Diamond Antifade Mountant (Thermo, P36961). The fluorescent images in the immuno-stained cells were captured using a confocal microscope and fluorescent microscope (Olympus FV-1000 and IX71, Tokyo, Japan). To count ciliated cells, more than 800 cells in five images per group were analyzed.

### 2.5. Scanning Electron Microscopy (SEM)

For scanning electron microscopy, samples were fixed with half Karnovsky’s solution (2.5% glutaraldehyde, 2% PFA in 0.1 M phosphate buffer pH 7.0) for 30 min at RT, followed by rinsing three times with 10% sucrose buffer. The samples were then post-fixed with an aqueous solution of 1% osmium tetroxide for 30 min and subsequently washed with water extensively. Then, they were dehydrated in a graded series of ethanol-water to 100% ethanol. After dehydration, samples were substituted in butyl alcohol, which is a medium for freeze-drying. The treated specimens were mounted on stubs with adhesive carbon tape and sputter-coated with a gold-palladium layer. Images were obtained with a JSM-6500 electron microscope (JEOL, Tokyo, Japan). 

### 2.6. Motility Analysis of the Ciliated Cells

For the analysis of ciliary beat frequency, a membrane with differentiated cells was cut from the transwell and placed on a slide with a coverslip set in the perfusion chamber. The chamber was mounted on a differential interference contrast (DIC) microscope (Olympus IX71, Tokyo, Japan). A movie of the ciliary beat was recorded by a high-speed camera (Prosilica GE-680, Allied Vision, Exton, PA, USA) at 157 frames per second and analyzed using TI Workbench software [14].

To analyze flow, fluorescent beads were added into the transwell chambers at 5 × 10^7^ beads/mL. The flow of fluorescent beads was recorded at 19 fps, and a total of 200 frames were acquired by using a time-lapse fluorescent microscope with a 20× objective. Stack images of the fluorescent beads were traced with a color footprint (Image J plug-in).

### 2.7. Western Blot Analysis

Cells were lysed with RIPA buffer (25 mM Tris-HCl pH 7.6, 150 mM NaCl, 1% NP-40, 1% sodium deoxycholate, 0.1% SDS) containing a protease inhibitor cocktail (Roche, 04693159001) and phosphatase inhibitor cocktail (Nacalai, 07575-61) on ice for 30 minutes. The soluble lysates were collected after centrifuge at 14,000 g for 10 min. Proteins in the cell lysates were separated by 4–20% gradient polyacrylamide gel and wet-transferred to PVDF membrane at 200 mA and 4 °C for 2 h. The membrane was blocked using Tris-buffered saline with Tween 20 (TBS-T) containing 2% bovine serum albumin, followed by the incubation with primary antibodies (Table S1) overnight at 4 °C. The membrane was then washed with TBS-T three times and incubated with secondary antibodies (Table S1) for 1 h at RT. Signals were developed using an enhanced chemiluminescence substrate (Nacalai, Chemi-Lumi One Super #02230), and images were acquired using an ImageQuant LAS 4000 detection system (GE Healthcare, Chicago, IL, USA).

### 2.8. Polymerase Chain Reaction (PCR)

Total RNA was extracted from cultured cells using the RNeasy Mini Kit (Qiagen, #74104, Germantown, MD, USA) according to the manufacturer’s instructions. The RNA concentration was measured using a NanoDrop ND-1000 spectrophotometer (NanoDrop Technologies, Wilmington, DE, USA). Double-stranded cDNA was synthesized from 2 μg of total RNA using a reverse transcription kit (Thermo, #4368813). Real-time quantitative PCR was performed using the FastStart Universal Probe Master kit (Roche, 04913957001) by StepOne (Applied Biosystems, Foster, CA, USA). A Roche Universal Probe #2 was used for *ATP5F1*, *DLL1*, *JAG1*, and *JAG2*. Probe #30 was used for *DLL4*. Probe #68 was used for *FOXJ1*. Relative RNA quantitation was performed using delta CT calculations. *ATP5F1* was used as a control. GoTaq Green Master Mix (Promega, #9PIM712, Wisconsin, USA) was used for analysis of *NOTCH1*, *NOTCH2*, and *NOTCH3* expression in FTECs. The DNA products were applied to a 1.5% agarose gel for quantification. Specific primer sequences are listed in Supplementary Table S2.

### 2.9. Statistical Analysis

Student’s *t*-test was used to compare the variation between two groups using GraphPad Prism software. When comparing three or more groups, we analyzed the data with the ANOVA test. All statistical calculations were performed on *n* = 3 or more. The values are expressed as the means ± SD. Significance levels were * *p* < 0.05, ** *p* < 0.01, and *** *p* < 0.001.

## 3. Results

### 3.1. Estrogen Regulates Ciliogenesis Through ERβ

**Figure 1:**
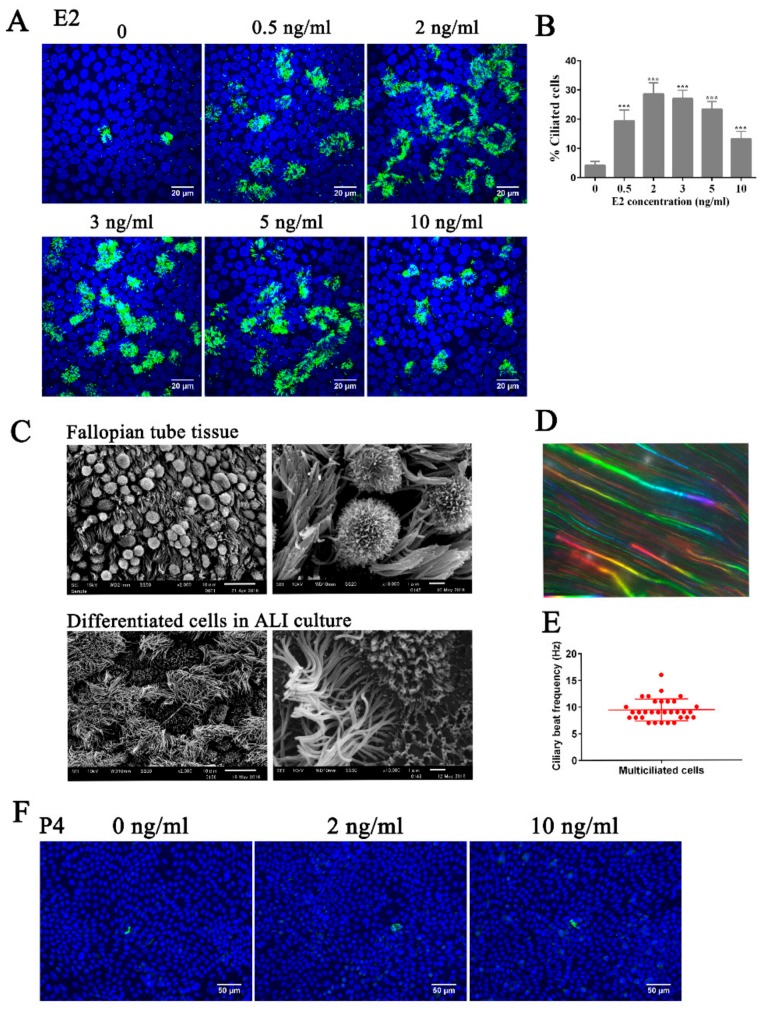
E2 is necessary and sufficient for ciliogenesis in fallopian tube epithelial cells (FTECs). (**A**) FTECs were cultured with different concentrations of E2 (0–10 ng/ml) in the basal medium. Cells on air–liquid interface (ALI) day 10 were stained for ac-tubulin (green) and nuclei (blue). Scale bars: 20 µm. (**B**) The number of ac-tubulin-positive cells in A was quantified (ANOVA test, *n* = 5, compared with the cells without E2). (**C**) SEM photomicrographs of the porcine fallopian tube (FT) tissue and the differentiated FTECs at ALI day10 incubated with 2 ng/mL E2. Scale bars: left panel, 10 μm; right panel, 1 μm. (**D**) This image represents stacked time-lapse pictures of the fluorescent beads, which were placed on the differentiated cells. (**E**) Ciliary beating frequency was measured using a high-speed camera. Thirty-two ciliated cells were analyzed. (**F**) FTECs were cultured with different concentrations of P4. Cells on ALI day 10 were stained for ac-tubulin (green) and nuclei (blue). Scale bars: 50 µm. Significance level: *** *p* < 0.001.

The fallopian tube mucosal environment is modulated by two steroids in the menstrual cycle: estrogen (E2) and progesterone (P4). In order to understand how these hormones regulate the differentiation of FTE, we established a primary culture of FTECs where ciliogenesis could be induced in ALI condition. When FTECs were grown in the presence of E2, MCCs were observed almost 10 days after induction. E2 is indispensable for ciliogenesis, as FTECs that grew in the absence of E2 displayed no or very little multiple cilia (Figure 1A). The optimal concentration of E2 for the most efficient ciliogenesis was 2 ng/mL (Figure 1A,B). SEM analyses showed that the differentiated FTECs displayed a morphology resembling incompletely the cytoarchitecture of FTE in vivo (Figure 1C). Furthermore, the FTECs showed vigorous ciliary motility, as revealed by the flow of fluorescent beads and direct captured using a high-speed camera (Figure 1D,E). Conversely, when we treated FTECs with P4 in lieu of E2, very few numbers of MCCs were induced (Figure 1F). These results indicate that E2 predominantly induces ciliogenesis, at least in vitro cultures.

**Figure 2:**
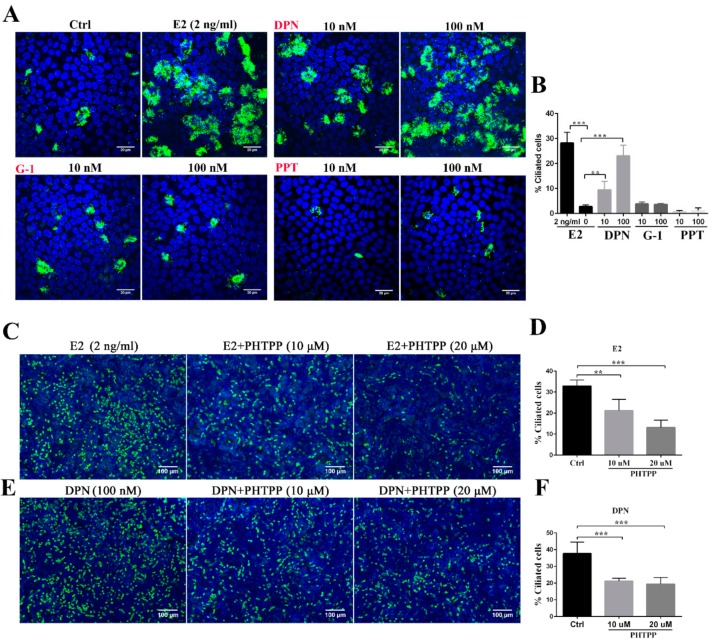
E2 promotes ciliogenesis through ERβ. (**A**) FTECs were incubated in the absence (Ctrl) and presence of E2, DPN, PPT and G-1. Cells on ALI day 15 were stained for ac-tubulin (green) and nuclei (blue). Scale bars: 20 µm. (**B**) The numbers of ac-tubulin-positive cells in A were quantified (ANOVA test, *n* = 5). (**C**–**F**) FTECs were cultured with E2 or DPN and with or without PHTPP. Cells on ALI day 15 were stained for ac-tubulin (green) and nuclei (blue). (**C**, **E**) The numbers of ac-tubulin-positive cells were quantified (**D**, **F**; ANOVA test, *n* = 5). Scale bars: 100 µm. Significance level: ** *p* < 0.01, and *** *p* < 0.001.

As a next step to dissect the molecular mechanism of ciliogenesis by E2, we focused on the identity of the estrogen receptors. There are two canonical signaling pathways for estrogen: one is mediated by steroid binding proteins, ERα and ERβ, and the other is through GPR30, one of the G-protein coupled receptors (GPCR) [15,16]. By using a specific agonist for each receptor, we could determine which receptor is responsible for E2-mediated ciliogenesis. Upon addition of DPN, a specific agonist for ERβ, to the FTEC culture, we could recapitulate the ciliogenesis as observed in E2 administration (Figure 2A,B). This is further reinforced by the administration of ERβ antagonist, PHTPP, in a dose-dependent manner (Figure 2C–F), because ciliogenesis did not reach a full-fledged state as observed in E2 or DPN. Meanwhile, the specific agonists for ERα and GPR30—PPT and G-1, respectively—did not show any obvious effects on ciliogenesis (Figure 2A,B). Collectively, we demonstrated that the effect of E2 on ciliogenesis was mediated by ERβ specifically.

### 3.2. Inhibition of the EGFR-MEK-ERK Pathway Promotes Ciliogenesis

**Figure 3:**
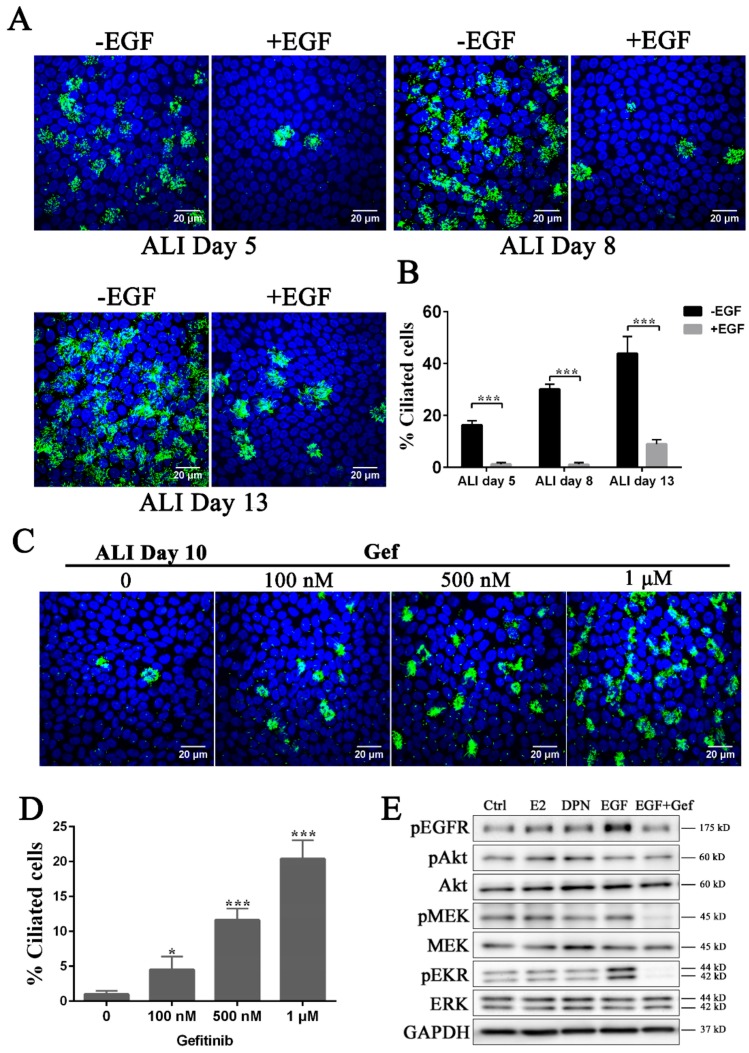
EGFR-MEK-ERK pathway negatively regulates ciliogenesis. (**A**) FTECs were cultured in basal medium with or without 10 ng/mL EGF, followed by the addition of 2 ng/mL E2 under ALI culture. Cells were stained for ac-tubulin (green) and nuclei (blue). Scale bars: 20 µm. (**B**) The numbers of ac-tubulin-positive cells in A were quantified (ANOVA test, *n* = 5). (**C**) FTECs were cultured with different concentrations of gefitinib with 10 ng/mL EGF. Cells on ALI day 10 were stained for ac-tubulin (green) and nuclei (blue). Scale bars: 20 µm. (**D**) The numbers of ac-tubulin-positive cells were quantified (ANOVA test, *n* = 5). (**E**) Western blot analysis of EGF-pathway related proteins in FTECs. FTECs P1 were thawed and passaged onto 12-well plate after confluency. In the last panel, cells were pre-treated with gefitinib for 30 min and then all FTECs were treated with other reagents for 15 min. Significance level: * *p* < 0.05, ** *p* < 0.01, and *** *p* < 0.001.

Epithelial cell differentiation is regulated by several growth factors. One of the important molecules for maintenance of the epithelial sheet is EGF [17]. While EGF exerts its effect mainly on cell proliferation, its role in ciliogenesis has remained an open question. In addition, in FTEC culture, ciliation takes almost two weeks after confluence. We assumed that EGF may attenuate the effects of E2 in ciliogenesis, as the basal medium used in the previous experiments included EGF. When we removed EGF from basal medium supplemented with E2, cilia started to form as early as ALI day 5 and increased on day 8–13 (Figure 3A,B). Even on day 15, FTEC culture showed a variable differentiation potential depending on the concentration of EGF (Figure S1A). This indicates that EGF weakens the effect of E2 on ciliogenesis. To further confirm the role of EGFR signaling in ciliogenesis, we used gefitinib, a selective inhibitor of the EGFR tyrosine kinase domain. Gefitinib was added to the basal medium without E2 from ALI day 0 to ALI day 15. On day 15, it promoted ciliogenesis in a concentration-dependent manner (100–1000 nM; Figure 3C,D). These results suggest that EGFR signaling is directly involved in the suppression of ciliogenesis. We further found that the phosphorylation of ERK, an EGFR-downstream protein, increased upon EGF treatment, and the activity of ERK and MEK, an upstream regulator of ERK, was inhibited by gefitinib. However, neither EGF nor gefitinib affected the level of phosphorylated AKT (Figure 3E and Figure S1B), indicating that the AKT-dependent pathway is not involved in ciliogenesis. Moreover, AKT, MEK, and ERK were not phosphorylated by E2 and DPN (Figure 3E and Figure S1B,C). These results collectively suggest that the EGF pathway suppresses ciliogenesis through the MEK and ERK pathway. As E2 and DPN did not affect the EGF pathway, the estrogen pathway might act in ciliogenesis independently from the EGF pathway. 

### 3.3. Estrogen and EGFR Pathways Regulate Notch Signaling in Ciliogenesis

**Figure 4:**
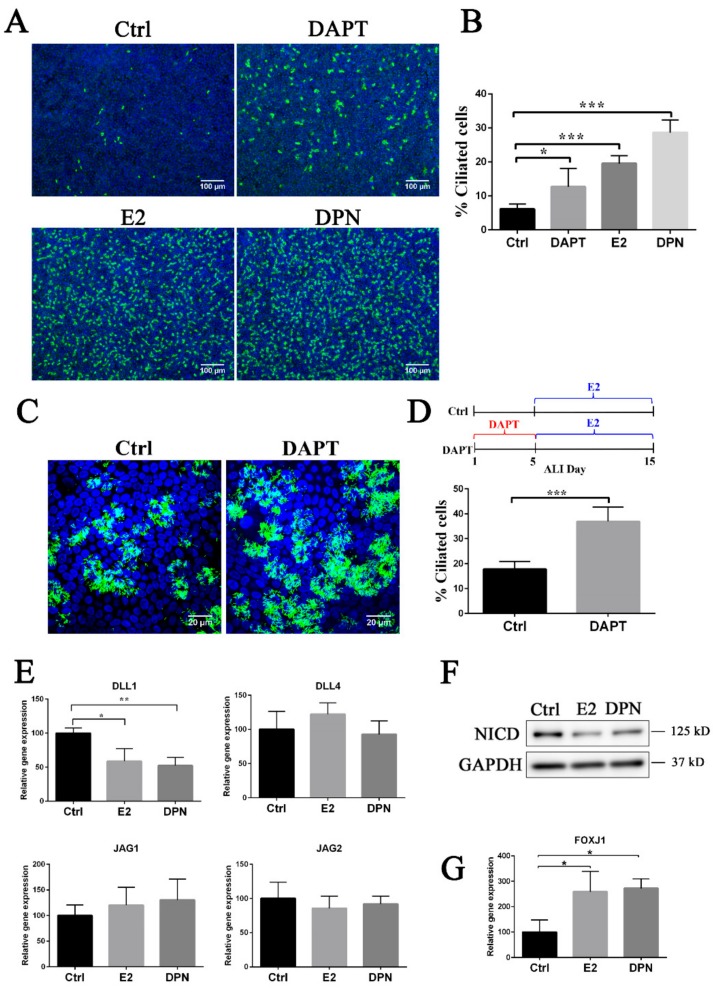
Estrogen regulated Notch signaling by suppressing DLL1 in ciliogenesis. (**A**) FTECs were cultured in basal medium (Ctrl) or with DAPT (10 μM), E2 (2 ng/ml), and DPN (100 nM) for ALI 10 days. Cells were stained for ac-tubulin (green) and nuclei (blue). Scale bars: 100 µm. (**B**) The numbers of ac-tubulin-positive cells were quantified (unpaired *t*-test, *n* = 7). **(C)** FTECs were cultured with or without DAPT (10 μM) for the first ALI 5 days, followed by E2 treatment (2 ng/mL) in both cells for another 10 days. Cells were stained for ac-tubulin (green) and nuclei (blue). Scale bars: 20 µm. (**D)** The numbers of ac-tubulin-positive cells were quantified (unpaired *t*-test, *n* = 7). (**E, G**). qRT-PCR for *DLL1*, *DLL4*, *JAG1*, *JAG2*, and *FOXJ1* in FTECs that were cultured for 24 h with or without 2 ng/mL E2 or 100 nM DPN (ANOVA test, *n* = 3). (**F**) Western blot analysis for NICD and GAPDH proteins in FTECs that were treated with E2 or DPN for 8 days in ALI. Significance level: * *p* < 0.05, ** *p* < 0.01, and *** *p* < 0.001.

The Notch pathway has been reported to negatively regulate ciliogenesis [13,18,19]. In Notch signaling, there are four subtypes of receptor: NOTCH1, NOTCH2, NOTCH3, and NOTCH4. *NOTCH1*, *NOTCH2*, and *NOTCH3* were expressed in porcine FTECs, but we could not detect the expression of *NOTCH4* (Figure S2A and data not shown). In order to obtain insight in the relationship between E2 and Notch signaling pathways, as well as to specify the molecular cascade, we attempted to inhibit Notch signaling by DAPT, a specific inhibitor of γ-secretase. We found that DAPT suppressed the expression of Notch intracellular domain (NICD), as well as Notch downstream genes *HES1* and *HEY1* (Figure S2B,C). Consistent with previous study, ciliogenesis was increased when FTECs were culture with 10 μM DAPT in basal medium. However, the effect of DAPT in inducing ciliogenesis was not as strong as E2 and DPN (Figure 4A,B). This suggests that Notch signaling affects the fate determination of FTECs, and estrogen signaling pathway may modulate it in either a direct or indirect manner. When FTECs were cultured in basal medium with DAPT for the first 5 days in an ALI condition and then grown in the presence of E2, they differentiated into MCC more drastically compared to the DAPT-untreated control (Figure 4C,D). In contrast, when cells were pre-treated with DAPT for either 3 or 10 days, there was no significant difference in differentiation (Figure S2D,E). This indicates that Notch acts as a gatekeeper to allow FTECs to differentiate into MCCs, but their competency might be restricted in specific time window. Since the estrogen pathway promoted ciliogenesis in FTECs (Figure 2), we assumed that E2 and DPN also attenuate Notch signaling to induce ciliogenesis. We then examined the effects of E2 and DPN on mRNA expression of Notch ligands, *DLL1*, *DLL4*, *JAG1*, and *JAG2*, 24 h after the administration of E2 or DPN. Both treatments significantly suppressed the expression of *DLL1* but hardly affected that of *DLL4*, *JAG1*, or *JAG4* (Figure 4E). The reduction of *DLL1* by E2 or DPN treatment in fact resulted in the downregulation of NICD cleaved from Notch1 (Figure 4F and Figure S2F). We also observed the upregulation of *FOXJ1*, a master regulator of ciliogenesis, upon treatment with E2 or DPN (Figure 4G). These results indicate that the estrogen pathway suppresses Notch signaling during the differentiation of FTECs into MCCs. 

**Figure 5:**
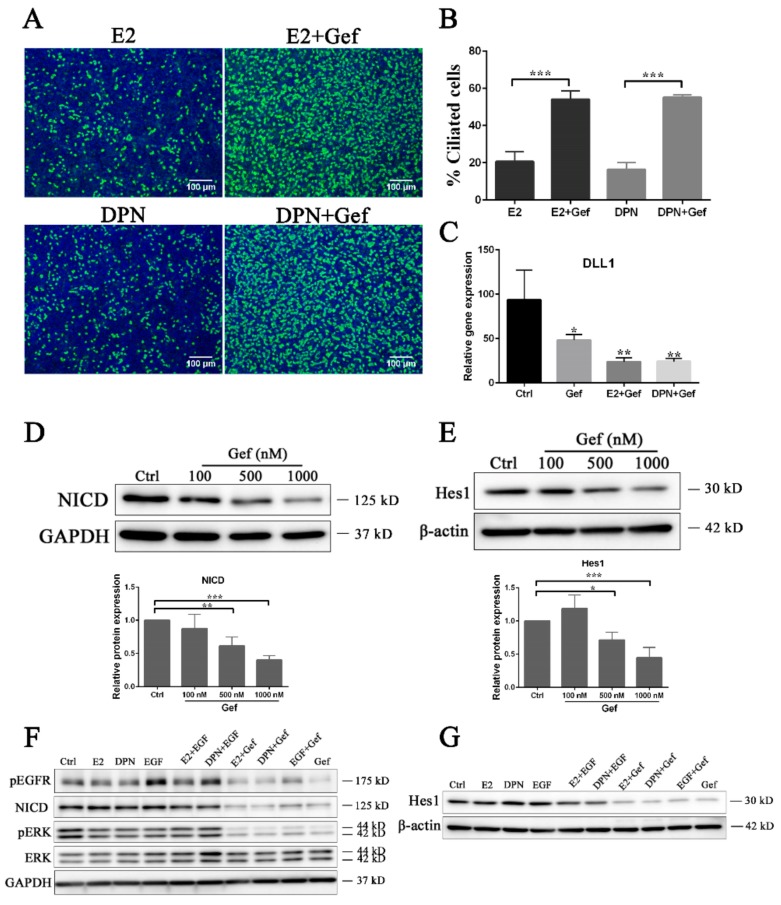
EGFR-MEK-ERK and estrogen pathways opposingly control Notch signaling through DLL1 to regulate ciliogenesis. (**A**) FTECs were incubated with E2 or DPN (2 ng/mL and 100 nM, respectively) and with or without 500 nM gefitinib (Gef) during the ALI culture. Cells on ALI day 10 were stained for ac-tubulin (green) and nuclei (blue). Scale bars: 100 µm. (**B**) The numbers of ac-tubulin-positive cells were quantified (Student’s *t*-test, *n* = 5). (**C**) qRT-PCR for *DLL1* in FTECs that were cultured with the reagents for 24 h (ANOVA test, *n* = 3). (**D**, **E**) Western blot analysis and quantification (*n* = 4) for (**D**) NICD and (**E**) Hes1 in FTECs that were cultured with gefitinib (0–1000 nM) for 4 days. (**F, G**) Western blot analysis for NICD, Hes1, and EGF pathway related proteins in FTECs that were cultured with indicated reagents for 4 days. Drug concentration: 2 ng/mL E2, 100 nM DPN, 10 ng/mL EGF, 500 nM gefitinib. Significance level: * *p* < 0.05, ** *p* < 0.01, and *** *p* < 0.001.

**Figure 6:**
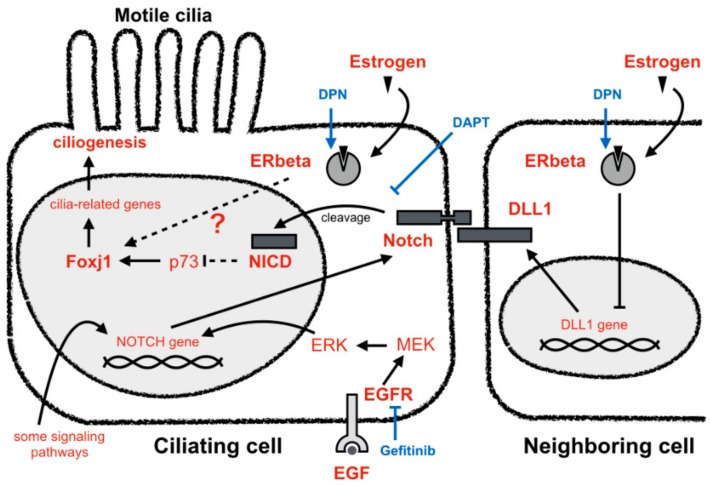
Molecular interplay between estrogen, EGF, and Notch signaling pathways in FTEC ciliogenesis. This schematic view illustrates the role of estrogen in multiple ciliogenesis of motile cilia. First, the activation of ERβ by estrogen suppresses *DLL1*, leading to the downregulation of Notch signaling in ciliating cells. FOXJ1 is a master regulator of multiple ciliogenesis, which is indirectly inhibited by Notch pathway. Therefore, inhibition of Notch pathway by estrogen facilitates the ciliogenesis of motile cilia. While there is no definitive evidence in this study, the possibility still exists that ERβ directly induces *FOXJ1*. Second, EGFR-MEK-ERK pathway stimulate Notch signal to maintain a proliferative state or induce differentiation into other cell types. Treatments with gefitinib or DAPT respectively suppress EGF and *NOTCH* pathway, and consequently induce ciliogenesis. There are several other pathways acting on the *NOTCH* gene. Ciliogenesis is established on the balance of many signaling machinery such as hormones, growth factors, and cell-to-cell communication.

In contrast, as EGF signaling suppresses ciliogenesis and is independent from the regulation by estrogen (Figure 3), we have questioned how EGF signaling regulates ciliogenesis in terms of the estrogen pathway. To address this, we administered E2 or DPN together with gefitinib to FTECs. Compared with the control (only with E2 or DPN treatment), ciliogenesis was dramatically promoted when gefitinib was combined with either E2 or DPN (Figure 5A,B). This suggests that the inhibition of EGFR is a synergistic pathway for the induction of MCCs together with the estrogen pathway. Since the Notch pathway is critical in regulating ciliogenesis, we finally examined the activation of Notch signaling upon the treatment of E2 or DPN and gefitinib. The combination of gefitinib with E2 or DPN more strongly inhibited *DLL1* mRNA expression than did single treatment with gefitinib (Figure 5C). This can explain why a higher proportion of ciliated cells was observed with gefitinib treatment (Figure 5A,B). We also found that gefitinib inhibited the expression of NICD as well as Hes1, a downstream target gene of Notch, in a concentration-dependent manner (Figure 5D,E and Figure S3A). Moreover, FTECs incubated with gefitinib showed the inactivation of EGFR and ERK, which was coincident with the suppression of NICD and Hes1 expression (Figure 5F,G; Figure S3B). The expression of NICD was unchanged in the presence of either E2 or DPN for at least 4 days but significantly reduced on ALI 8 days (Figure 5F and Figure 4G). Taken together, the estrogen and EGF pathways regulate Notch signaling in opposite ways in terms of ciliogenesis in FTECs (Figure 6). 

## 4. Discussion

While several previous reports have clarified the roles of sex steroids in the differentiation of FTECs, there has been no direct evidence regarding ciliogenesis. Moreover, the molecular mechanisms underlying the regulation of ciliogenesis in the female reproductive tract have remained enigmatic. In this study, we uncovered the role of the estrogen pathway in the differentiation of FTECs into MCCs. Several previous studies have shown that ERα is not required for FTEC ciliogenesis [20], implying the involvement of another subtype. Our study concluded that ERβ is responsible for ciliogenesis. Although the administration of E2 or DPN eventually resulted in *DLL1* downregulation and *FOXJ1* upregulation prior to the generation of motile cilia in cultured FTECs, we could not determine whether this directly acted on their transcription. The downstream genes directly regulated by ERβ for ciliogenesis should be clarified in future study.

Our study also suggests that EGF coordinates with the Notch signal through the activation of the EGFR-MEK-ERK pathway to control the number of MCCs (Figure 6). Administration of EGF results in the proliferation of Goblet cells by increasing the expression of the major secretory molecule, mucin MUC5AC, in the tracheal epithelium [21,22]. Moreover, in the human FT, EGF promotes the cell proliferation while inhibition of Notch drove the production of MCCs [13]. Although both EGF and Notch signaling pathways are involved in proliferation and cell-fate determination [23,24,25], the regulation is context-dependent and interrelated. In order to clarify the molecular mechanism underlying ciliogenesis via cooperation of EGF and Notch, we must establish a more explicit method to dissect the cell-to-cell interaction related to the Notch pathway. 

We suggest that the estrogen pathway might act in ciliogenesis independently from the EGF pathway, as treatment of FTECs with E2 or DPN did not show any significant change in the EGF signaling pathway. Alternatively, EGF may suppress the activation of ERβ to inhibit ciliogenesis and activate the ERα pathway. Several reports have indicated that ERα functions in an opposing manner to ERβ in regulating the cell cycle, sensitivity to tumor necrosis factor α (TNFα), and osteogenesis [26,27,28]. In our study, E2 promoted ciliogenesis in a concentration-dependent manner, up to 2 ng/mL. Ciliogenesis decreased with higher concentrations of E2 (5-10 ng/ml), suggesting the possibility that a high concentration of E2 activates ERα rather than ERβ. Since EGF can induce the phosphorylation of ERα [29,30,31], we assume that EGF inhibits the ERβ pathway via the activation of ERα to attenuate ciliogenesis. To clarify this, we should analyze the activation of ERα and ERβ with EGF or gefitinib. 

Increasing lines of recent evidence have shown that FTECs may be an origin of HGSC [10,11,12]. In general, the EGF, Notch, and estrogen pathways are known to be key regulators for cell proliferation and carcinogenesis [32,33,34]. For example, EGFR is enriched in airway basal cells rather than in differentiated cells [24]. When inhibiting EGFR with gefitinib, the terminal differentiation of progenitor cells is facilitated [24]. Stem/progenitor cells act as a functional niche that continuously provides Notch ligands to surrounding daughter secretory cells. Without these ligands, the progenitor cell pool fails to survive, resulting in the depletion of stem cells [35,36]. Therefore, we suppose that E2 also inhibits *DLL1* secreted by neighbor cell niche, resulting in the suppression of the Notch pathway. The estrogen and EGFR pathway function oppositely in ciliogenesis. Furthermore, the EGFR inhibitor gefitinib seems to have a stronger inhibition effect on Notch signals than estrogen and DPN. However, estrogen and DPN could induce more cilia when compared with the single treatment of gefitinib. This indicates that the estrogen pathway may also have another role in ciliogenesis in addition to the inhibition of *DLL1*. Moreover, efficient ciliogenesis might need other inputs besides the reduction of Notch signaling.

Since Notch is an important regulator of stemness and differentiation of epithelial cells, our results provide an important toehold regarding the cellular mechanisms that maintain homeostasis of the FTE. Considering the roles of E2, EGF, and Notch involved in the regulation of FTE homeostasis, dysfunction of these factors may transform the FTECs into tumor cells, which are the basis for HGSC. It is well known that ERα and EGF play an important role in breast cancer [31,37]. In contrast, relatively little is known about the relationship between ovarian carcinomas and the estrogen pathway. Since E2 suppressed *DLL1* and promoted ciliogenesis via ERβ, our next step is to test whether ERα, together with EGF, contributes to the early stage of carcinogenesis in FTECs.

## Supplementary Materials

The following are available online at https://www.mdpi.com/2073-4409/8/8/933/s1, Figure S1: EGF inhibited ciliogenesis and downregulated phosphorylation of EGFR-MEK-ERK pathway, Figure S2: Effects of DAPT on ciliogenesis, Figure S3: Characterization of anti-NICD antibody by Western blotting, Table S1: Antibodies, Table S2: Primer sequences.
