# Supplementary material for: Estrogen and EGFR Pathways Regulate Notch Signaling in Opposing Directions for Multi-Ciliogenesis in the Fallopian Tube
Source: Cells. 2019 Aug 19;8(8):933. doi: 10.3390/cells8080933 (PMC6721734; doi:10.3390/cells8080933)
Supplement: Supplementary file 1 [file cells-08-00933-s001.pdf]

Supplementary Materials

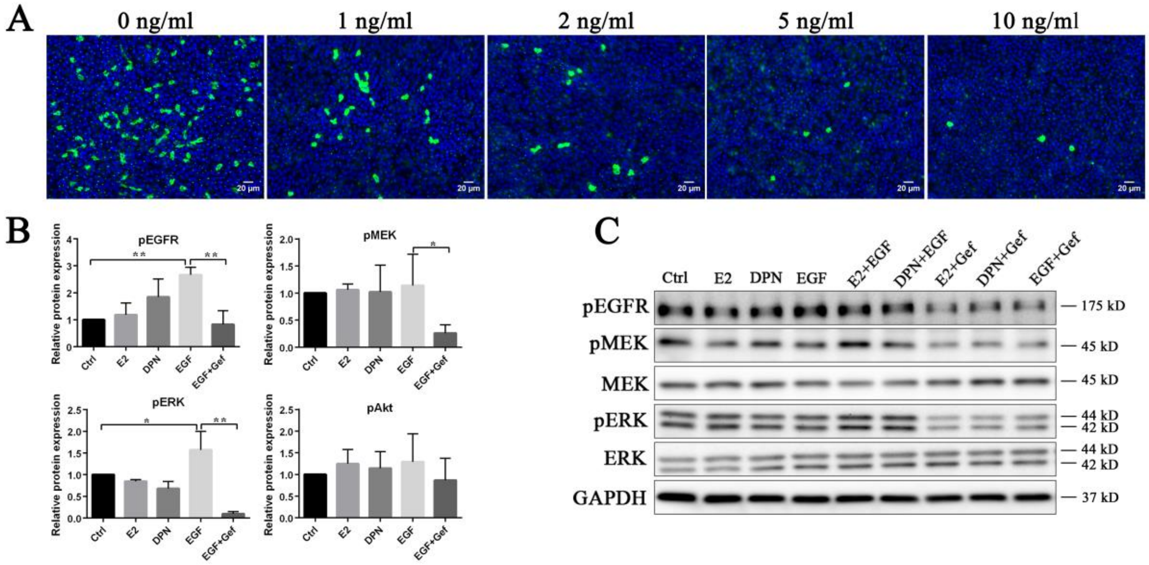

Figure S1 EGF inhibited ciliogenesis and downregulated phosphorylation of EGFR-MEK-ERK pathway.

A. FTECs were cultured in the presence of E2, followed by the addition of 0-10 ng/ml EGF under ALI culture. Cells on ALI day 15 were stained with ac-tubulin (green) and DAPI (nuclei, blue). Scale bars: 20 μm. **B.** Quantification of relative protein expression in figure 3E (ANOVA test, n = 3). **C.** Gefitinib inhibited the phosphorylation of EGFR, MEK, and ERK. Western blots of whole-cell lysates from FTECs after treatment with various additives for 24 h. Concentrations for each compound were 2 ng/ml for E2, 100 nM for DPN, 10 ng/ml for EGF, and 500 nM for gefitinib.

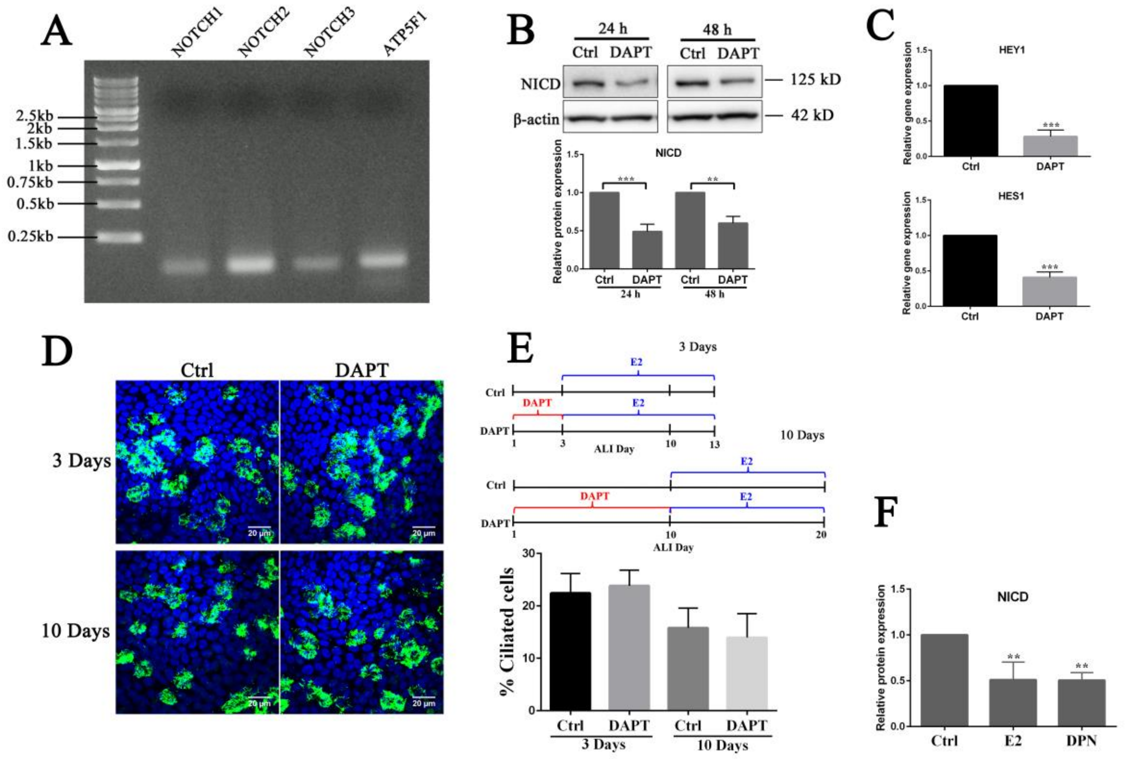

Figure S2 Effects of DAPT on ciliogenesis.

A. Analysis of NOTCH1, NOTCH2 and NOTCH3 expression in FTECs. ATP5F1 was used as a positive control. **B.** Western blot analysis for NICD expression. Whole cell lysate was prepared from FTECs cultured with or without DAPT (10 μM) in basal medium for 24 and 48 hours. β-actin was used as an internal control (unpaired *t*-test, n = 3, compared to the Ctrl group). **C.** qRT-PCR for analysis of HES1 and HEY1 expression in FTECs that were cultured for 24 h with or without DAPT (10 μM) (unpaired *t*-test, n = 3, compared to the Ctrl group). **D.** FTECs were pretreated with or without DAPT (10 μM) for either 3 or 10 days, followed by withdrawal of DAPT and addition of 2ng/ml E2 for another 10 days. Cells were stained with anti-ac-tubulin (green) antibody and DAPI (nuclei, blue). Scale bars: 20 μm. **E.** Time course for drug treatment in FTECs culture. Quantification of ac-tubulin positive cells. (unpaired *t*-test, n = 7). **F.** Relative protein expression quantified from figure 4G (ANOVA test, n = 3, compared to the Ctrl group).

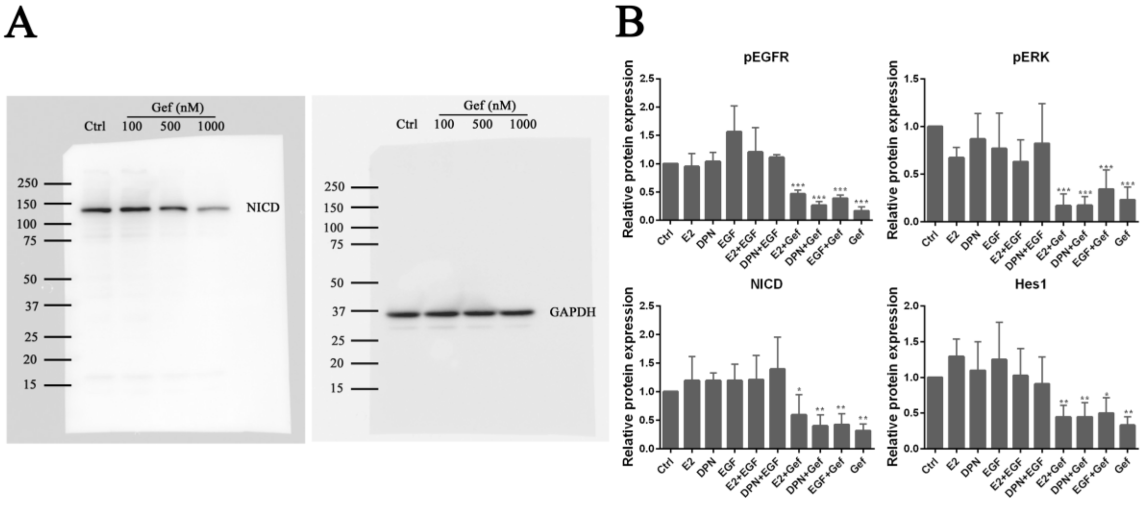

Figure S3 Characterization of anti-NICD antibody by Western blotting.

A. Full image of PVDF membrane probed with anti-NICD antibody. Whole cell lysate was prepared from FTECs cultured with gefitinib (0-1000 nM) for 4 days. GAPDH was used as an internal control. **B.** Relative protein expression quantified from figure 5F, G (ANOVA test, n = 3, compared to the Ctrl group).

Table S1: Antibodies.

Name	NO.	Company	Country
mouse anti-acetylated α-tubulin	6-11B-1	Sigma	Saint Louis, USA
mouse anti-GAPDH	60004-1-Ig	Proteintech	Rosemont, USA
rabbit anti-phospho-EGFR	3777S	CST	Danvers, USA
rabbit anti-ERK	9102	CST	Danvers, USA
rabbit anti-phospho-ERK	9101S	CST	Danvers, USA
rabbit anti-MEK	9122	CST	Danvers, USA
rabbit anti-phospho-MEK	9121S	CST	Danvers, USA
rabbit anti-Notch1	ab52627	Abcam	Cambridge, UK
rabbit anti-Hes1	GTX108356	GeneTex	California, USA
rabbit anti-β-actin	20536-1-AP	Proteintech	Danvers, USA
HRP-conjugated anti-mouse IgG	7076	CST	Danvers, USA
HRP-conjugated anti-rabbit IgG	7074	CST	Danvers, USA
Alexa Fluor 488 conjugated anti-mouse IgG	A11029	Invitrogen	California, USA
Alexa Fluor 488 conjugated anti-rabbit IgG	A11008	Invitrogen	California, USA
Alexa Fluor 568 conjugated anti-mouse IgG	A11004	Invitrogen	California, USA
Alexa Fluor 568 conjugated anti-rabbit IgG	A11036	Invitrogen	California, USA

Table S2: Primer sequences

Gene name		Sequence
**ATP5F1**	Sense	5'- CACGTGGTGCAGAGCATC-3'
	Antisense	5'-TCTTTGCGAGCAGCTTTAGA-3
**DLL1**	Sense	5'-GAATGGAGGGAGCTGCAC-3'
	Antisense	5'-CACTCACGCAGATCCT-3'
**DLL4**	Sense	5'-GTGGTGCTGGTGGTACTGTG-3'
	Antisense	5'-AGTCCGACAAGTTGTTCATGG-3'
**JAG1**	Sense	5'-CTCACAGCTATGCAAACACCA-3'
	Antisense	5'-CCTAAGACTGCATCACCATCTG-3'
**JAG2**	Sense	5'-GCCCAATCCCTGTGTGAA-3'
	Antisense	5'-GGTATTGTGCGTGCAGGTT-3'
**FOXJ1**	Sense	5'-AAACAGACGCTGCCCAAG-3'
	Antisense	5'-AAGTTGCCTTTGAGGGGTTC-3'
**NOTCH1**	Sense	5'-TGGATGGCATCAATTCCTTTT-3'
	Antisense	5'-CGTCATGCTGGCAGTAGC-3'
**NOTCH2**	Sense	5'-GGAGGTTCCTGTATCGATGG-3'
	Antisense	5'-ACACTTGTCCCCAATGAAGC-3'
**NOTCH3**	Sense	5'-AAGTCCATTTCATTCTCTCCATTC-3'
	Antisense	5'-GATGACCTCTTGGTTGCACTG-3'
**NOTCH4**	Sense	5'-GTGGTGCTGGTGGTACTGTG-3
	Antisense	5'-AGTCCGACAAGTTGTTCATGG-3
**HEY1**	Sense	5'-GCCGTCGAGACCGGATCAATAA-3'
	Antisense	5'-GCATTCCCGAAACCCCAAACTC-3'
**HES1**	Sense	5'-CTAAGCACAGACCCGAGCGT-3'
	Antisense	5'-GGTCATGGCGTTGATCTGGG-3'
